# Integrated metabolomics and network pharmacology to decipher habitat-driven metabolic diversity and *α*-glucosidase inhibitory mechanisms in guava leaves

**DOI:** 10.1016/j.fochx.2026.104274

**Published:** 2026-08-03

**Authors:** Xin-Yu Tao, Zhou-Wei Wu, Xiao-Cui Liu, Chen-Xi Quan, Marco Antonio Cabero, Ming-Hua Qiu

**Affiliations:** a State Key Laboratory of Phytochemistry and Natural Medicines, Kunming Institute of Botany, Chinese Academy of Sciences, Kunming 650201, Yunnan, China; b University of Chinese Academy of Sciences, Beijing 100049, China; c National Museum of Natural History, La Paz, Bolivia; d Andean Road Countries for Science and Technology (ARCST), La Paz, Bolivia

**Keywords:** *Psidium guajava*, Metabolic diversity, Phenolic chemotypes, Widely targeted metabolomics, *Α*-Glucosidase inhibition, Network pharmacology

## Abstract

Guava (*Psidium guajava* L.) leaves are valuable functional ingredients; however, evidence linking environmental origin to metabolite-resolved bioactivity remains limited. This study investigated leaves from five Bolivian sites across two ecological regions. While the total phenolic content remained stable, the total flavonoid content and *α*-glucosidase inhibition varied significantly across origins. Widely targeted LC–MS/MS annotated 1230 phenolic features and 753 differential metabolites, revealing an environmental-driven metabolic trade-off: high-irradiance lowlands accumulate lipophilic aglycones, whereas rainforests sequester hydrophilic flavonoid glycosides. Chemometrics revealed origin-dependent chemotypes: Amazonian extracts are glycoside-rich with strong enzymatic inhibition, whereas Lowland extracts are aglycone-enriched for radical scavenging. Network pharmacology and molecular docking prioritized norwogonin, predicting high TNF affinity (−10.1 kcal/mol) and AGE–RAGE axis targeting (e.g., AGER, AKT1, MAPK14). These predictions require experimental validation. Resolving the chemical heterogeneity of Bolivian guava, this work proposes a dual-chemotype classification, providing a robust framework for the strategic sourcing and standardization of functional food resources.

## Highlights

• Widely targeted metabolomics annotated 1230 phenolics in Bolivian guava leaves.
• Habitat drives a metabolic trade-off between aglycones and flavonoid glycosides.
• Lowland-enriched norwogonin was identified as a highly potent bioactive marker.
• Network pharmacology revealed AKT1 and AGER as core targets in the AGE-RAGE axis.
• A dual-chemotype framework was established to guide strategic botanical sourcing.

## Graphical abstract

**Figure:**
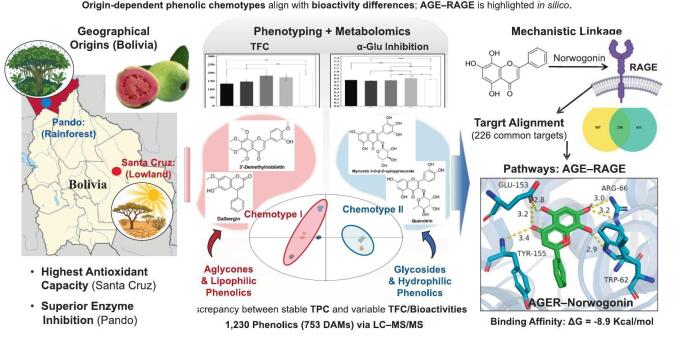

## 1 Introduction

Dietary management of postprandial glucose represents a critical cornerstone of modern metabolic health paradigms. Inhibiting *α*-glucosidase via dietary polyphenols is a well-recognized strategy to modulate glucose release from carbohydrates, thereby suppressing the downstream formation of advanced glycation end products (AGEs) (El-Saadony et al., 2024). These accumulated AGEs engage their specific cell-surface Receptor for Advanced Glycation End Products (RAGE), triggering sustained NF-*κ*B-dependent cellular activation, oxidative stress, and inflammatory cascades that drive downstream vascular and metabolic dysfunctions (Schmidt et al., 2001). Given that the chronic activation of the AGE–RAGE axis serves as a primary driver of metabolic complications, incorporating bioactive-rich botanical ingredients into functional food systems offers a promising preventive approach to mitigate these pathological cascades (Freitas et al., 2024). Consequently, identifying phenolic-rich materials capable of simultaneously hindering enzyme activity and attenuating downstream AGE–RAGE signaling is highly valuable for the development of targeted functional foods.

*Psidium guajava* L. (guava) is a globally significant fruit crop of substantial economic and agricultural value. While the fruit is cultivated primarily for fresh consumption, the leaves constitute a severely underutilized biomass source (Gutiérrez et al., 2008). Recently, guava leaves have gained considerable traction as functional tea substitutes and natural hypoglycemic agents within the global beverage and nutraceutical industries (Butt et al., 2025). These functional benefits are largely attributed to an abundance of phenolic acids and flavonoids characterized by high structural diversity (Shen et al., 2022). However, the large-scale industrial application of guava leaves is hindered by significant batch-to-batch variations in their functional potency. This inconsistency arises because plant secondary metabolites are highly sensitive to habitat-specific stressors, and crude, macro-spectrophotometric metrics inherently fail to reflect molecule-specific bioactivities (Raposo et al., 2024). Consequently, metabolite-resolved, high-resolution evidence linking geographical provenance to functional quality remains elusive.

Secondary metabolites are not static markers; rather, they represent dynamic phenotypic adaptations shaped by macroclimatic and micro-environmental stressors. Emerging evidence suggests that climatic variables, including solar UV radiation, humidity, and altitudinal gradients, significantly alter the polyphenolic accumulation profiles of *P. guajava* (El-Feky & AbdelRahman, 2025; Majhi et al., 2023; Usman et al., 2022). However, it remains poorly understood whether such environmental divergence consistently translates into reproducible, origin-specific chemotypes. Identifying potential “metabolic trade-offs”, such as the environmentally-driven shifts between lipophilic aglycones synthesized for photoprotection in high-irradiance zones and hydrophilic glycosides sequestered for stress resilience in humid environments, is essential for establishing a robust molecular framework for the geographical authentication and quality grade-valuation of guava-derived resources.

Bolivia encompasses an extraordinary degree of agro-ecological heterogeneity, characterized by a sharp bioclimatic gradient between the semi-arid tropical lowlands of Santa Cruz and the hyper-humid Amazonian rainforest of Pando (Paniagua-Zambrana et al., 2020). This ecological contrast provides a compelling natural laboratory to examine how distinct habitat-specific stressors drive the metabolic divergence of specific bioactive clusters. Although widely targeted metabolomics represents the gold-standard approach for revealing region-associated chemotype patterns in various botanical species (Liang et al., 2024; Younis et al., 2025; Zeng et al., 2025), empirical evidence integrating this profound ecological diversity with South American *P. guajava* phenolic chemotypes and their specific *α*-glucosidase inhibitory mechanisms has yet to be systematically established.

To address these critical knowledge gaps, the present study integrates high-coverage LC–MS/MS metabolomics (annotating 1230 features) with functional enzymatic screening and in silico computational modeling across multiple geographical accessions to: (i) resolve habitat-specific phenolic fingerprints and establish a dual-chemotype classification framework to facilitate raw material standardization; (ii) elucidate the environmentally-driven metabolic trade-offs and the resulting variations in *α*-glucosidase inhibitory (AGI) potency; and (iii) prioritize potent bioactive markers (notably norwogonin) and construct mechanistic hypotheses linking chemical provenance to the AGE–RAGE axis (targeting AGER, RELA, TNF, AKT1, and MAPK14) via network pharmacology and molecular docking. By bridging the gap between macro-ecological heterogeneity and metabolite-resolved bioactivity, this study delivers a robust, science-based framework for the strategic geographical sourcing, functional standardization, and high-value valorization of guava leaves as industrial ingredients.

## 2 Materials and methods

### 2.1 Chemicals and reagents

Gallic acid (≥98%), rutin (≥98%), Trolox (≥97%), acarbose (≥95%), Folin–Ciocalteu reagent, and *α*-glucosidase (from *Saccharomyces cerevisiae*) were purchased from Sigma–Aldrich (St. Louis, MO, USA). LC–MS–grade methanol (≥99.9%), acetonitrile (≥99.9%), isopropanol (≥99.9%), formic acid (≥99%), and acetic acid (≥99.7%) were obtained from Merck (Darmstadt, Germany). Commercial kits used for total phenolic content (TPC), total flavonoid content (TFC), and antioxidant capacity (ABTS) assays were sourced from Beyotime Biotechnology (Shanghai, China).

### 2.2 Sample collection

Fresh, mature *P. guajava* leaves were collected between February and April 2024 from five representative sites across two distinct agro-ecological regions in Bolivia: the semi-arid tropical lowlands of Santa Cruz (Sites A1, B1) and the humid Amazonian rainforest of Pando (Sites C1, F1, P1) (Table 1). To ensure representative sampling, leaves were harvested from at least ten randomly selected trees at each site.

**Table 1:** Sampling sites and geographical parameters.

Sample ID	Location Description	Latitude (°)	Longitude (°)	Elevation (m)	Agro-ecological zone
A1	San Ramón, Santa Cruz, Bolivia	16.6156° S	62.4960° W	277	Tropical lowland
B1	San Ramón, Santa Cruz, Bolivia	16.6118° S	62.5000° W	268	Tropical lowland
C1	Cobija, Pando, Bolivia	11.0267° S	68.7692° W	280	Amazonian rainforest
F1	Filadelfia, Pando, Bolivia	11.3733° S	68.9027° W	183	Amazonian rainforest
P1	Porvenir, Pando, Bolivia	11.2372° S	68.7036° W	222	Amazonian rainforest

**Note:** Agro-ecological zones were classified based on regional climatic and environmental characteristics.

Three independent biological replicates (*n* = 3) were prepared for each site; each replicate consisted of equal fresh mass pooled from at least ten individual trees to mitigate intra-site variability. Upon collection, samples were immediately snap-frozen in liquid nitrogen, vacuum-sealed and then stored at −80 °C.

### 2.3 Determination of phenolic components and bioactivities

To ensure a direct and robust comparison between chemical profiles and functional readouts, a standardized hydroethanolic extract was prepared for all spectrophotometric assays. Briefly, ground dried leaves (0.03 g, 40–60 mesh) were extracted with 1.5 mL of 60% (*v*/v) aqueous ethanol at 60 °C for 2 h. After centrifugation (12,000 rpm, 10 min, 4 °C), the supernatant was collected. For each biological replicate, extraction and assays were performed independently in triplicate to ensure statistical power.

#### 2.3.1 Determination of TPC and TFC

The total phenolic content (TPC) was determined using the Folin-Ciocalteu colorimetric method (Ainsworth & Gillespie, 2007; Singleton & Rossi, 1965). This reaction mixture was incubated in the dark for 30 min and the absorbance was measured at 765 nm. Results were expressed as mg gallic acid equivalents per gram dry weight (mg GAE/g DW). The total flavonoid content (TFC) was evaluated via the aluminum chloride (AlCl₃) colorimetric assay, with measurements conducted at 510 nm (Zhishen et al., 1999). Results were expressed as mg rutin equivalents per gram dry weight (mg RE/g DW).

#### 2.3.2 Antioxidant capacity assays

The antioxidant capacity was determined through the ABTS•^+^ radical cation decolorization assay, with the decrease in absorbance monitored at 734 nm (Re et al., 1999). The ABTS•^+^ solution was prepared by reacting 7 mM ABTS with 2.45 mM potassium persulfate and diluted to an absorbance of 0.70 ± 0.02 at 734 nm. After 6 min of reaction with the sample, the absorbance was measured. Trolox was employed as the reference standard, and results were expressed as mmol Trolox equivalents per gram dry weight (mmol TE/g DW).

#### 2.3.3 Determination of α-glucosidase inhibitory activity

The *α*-glucosidase inhibitory potential was determined at a fixed final concentration (200 μg/mL) using *p*-nitrophenyl-*α*-D-glucopyranoside (pNPG) as the substrate. The enzymatic reaction was monitored at 405 nm (Apostolidis & Lee, 2010). Acarbose (50 nM) was employed as the positive control. Inhibition was calculated as a fraction (0–1) relative to the blank control.

$$\text{Inhibition}\;\left(\text{fraction}\right)=\frac{{\mathrm{\Delta A}}_{\text{control}}-{\mathrm{\Delta A}}_{\text{sample}}}{{\mathrm{\Delta A}}_{\text{control}}-{\mathrm{\Delta A}}_{\text{blank}}}$$

where Δ*A*_control_, Δ*A*_sample_, and Δ*A*_blank_ represent the absorbance changes of the control, test sample, and sample blank, respectively.

### 2.4 Widely-targeted metabolomics profiling of *P. guajava*

#### 2.4.1 Sample preparation

The 60% ethanolic extracts obtained in Section 2.3 were filtered through 0.22 μm membranes into vials prior to LC–MS/MS analysis. To ensure analytical stability, internal standards were added at the extraction stage, and quality control (QC) samples were prepared by pooling equal aliquots from all samples to monitor instrument drift and ensure data reproducibility throughout the analytical sequence.

#### 2.4.2 UPLC-MS/MS analysis

Chromatographic separation was performed on a Vanquish UHPLC system (Thermo Fisher Scientific) equipped with a Kinetex C18 column (2.1 × 50 mm, 2.6 μm) maintained at 25 °C. The mobile phase was 0.01% aqueous acetic acid (A) and acetonitrile/isopropanol (1:1, *v*/v) (B). The 20-min gradient program (5%–95% B) operated at 0.3 mL/min with a 1 μL injection volume and an autosampler temperature of 4 °C. Pooled QC samples were injected every 10 runs to ensure system stability.

MS detection utilized a Stellar linear ion trap mass spectrometer (Thermo Fisher) equipped with an H-ESI source operating in parallel reaction monitoring (PRM) mode. Ion source parameters were as follows: spray voltage 3.8 kV (positive mode) and − 3.4 kV (negative mode); sheath, auxiliary and sweep gas at 50, 15 and 1 arb units, respectively; ion transfer tube temperature of 320 °C, and a vaporizer temperature of 450 °C.

For metabolite annotation, raw LC-MS/MS data were interrogated against a proprietary in-house database alongside extensive public repositories, such as MassBank, GNPS, MoNA, and mzCloud. The identification process employed a tiered approach: first, filtering by accurate mass with a tolerance of <5 ppm, followed by a rigorous comparison of secondary fragment ions. An MS/MS similarity score threshold was employed to confirm the spectral matching. This workflow ensured that all prioritized phenolic markers were annotated as Level 2 (putatively annotated compounds) according to MSI standards.

### 2.5 Statistical analysis of chemical and bioactivity data

All data (*n* = 3) are presented as mean ± SD. Significant differences among sampling sites were evaluated by one-way ANOVA with Tukey's post hoc test (*p* < 0.05) using SPSS Statistics 27. Relationships between phytochemical composition and bioactivity (*n* = 15) were assessed using Pearson and Spearman rank correlations across 1230 phenolic features. The Benjamini–Hochberg procedure was applied to control the false discovery rate (FDR). The top 20 metabolite features for each endpoint were prioritized based on adjusted *p*-values.

### 2.6 Statistical analysis of metabolomics data

Metabolomic data were normalized using internal standards, filtered for missing values (> 20%), and imputed using the k-nearest neighbors (KNN) algorithm in MetaboAnalyst 6.0 (Pang et al., 2024). Following log-transformation and auto-scaling, unsupervised principal component analysis (PCA) and orthogonal partial least squares discriminant analysis (OPLS-DA; validated by 7-fold cross-validation and 1000 permutations) were performed. Differentially accumulated metabolites (DAMs) were identified based on the combination of Variable Importance in Projection (VIP) values >1.0 from the OPLS-DA model with a two-sided *t*-test with FDR < 0.05 and |log₂FC| ≥ 1.0. Hierarchical clustering analysis (HCA) was performed using Euclidean distance and Ward's linkage. To distinguish the biological importance of identified DAMs across the 10 pairwise comparisons, a post-statistical tiered prioritization system was implemented: Tier 1 (broad-spectrum core markers) required significance in ≥3 comparisons, core status in ≥1 comparison, and max (VIP) ≥ 1.5; Tier 2 (high-impact, comparison-specific markers) included DAMs significant in 1–2 comparisons with either max (|log_2_FC|) > 3 or max (VIP) ≥ 2.0; and Tier 3 (supportive markers) comprised remaining core features or those with max (VIP) ≥ 1.5. These tiers represent statistical prioritization only and are independent of MS/MS annotation confidence, as detailed in Supplementary Methods Section 1.

### 2.7 Network pharmacology analysis

To ensure the reproducibility and traceability of the network pharmacology framework, all bioinformatic data retrieval, target mapping, and functional enrichment analyses were uniformly executed in November 2025. To identify protein targets of the bioactive phenolics, SMILES strings were obtained from PubChem (Kim et al., 2023) and analyzed via SwissTargetPrediction (*Homo sapiens* setting) (Daina et al. Yamagishi, 2019), retaining only targets with non-zero probability. Predicted compound–target pairs and filtered target lists are provided in Supplementary Data S4. Disease-related targets associated with oxidative stress and diabetes were acquired from GeneCards (Safran et al., 2021) (relevance score ≥ 5) and OMIM databases (Amberger et al., 2019). The intersection between compound and disease targets was visualized using a Venn diagram in Cytoscape 3.10.2 (Shannon et al., 2003). The disease target set and the overlapping targets used for downstream analyses are listed in Supplementary Data S3.

A human PPI network was constructed using STRING (Szklarczyk et al., 2025) with a minimum confidence score of 0.400. Core targets were identified in Cytoscape based on degree, betweenness, and closeness centrality metrics exceeding the network medians. Topology metrics and selected core nodes are provided in Supplementary Data S5. Functional enrichment was performed using DAVID (Dennis et al., 2003), incorporating Gene Ontology (GO) (Aleksander et al., 2023) and Kyoto Encyclopedia of Genes and Genomes (KEGG) pathways (Kanehisa & Goto, 2000). Statistical significance was defined by a Benjamini–Hochberg adjusted *p* < 0.05. Full GO and KEGG outputs are provided in Supplementary Data S6.

### 2.8 Molecular docking validation

Molecular docking was conducted to assess predicted binding affinities between phenolic markers and core targets (AGER, RELA, TNF, AKT1, and MAPK14). Ligand structures from PubChem were energy-minimized using the MMFF94 force field in Chem3D (Halgren, 1996), and prepared in AutoDock Tools 1.5.6 by adding Gasteiger charges and defining rotatable bonds (Morris et al., 2009). Protein crystal structures were retrieved from the RCSB Protein Data Bank (Berman et al., 2000) and prepared by removing water molecules and co-crystallized ligands.

Docking was performed using AutoDock Vina 1.1.2, with an exhaustiveness of 32 to ensure robust conformational sampling. The pose with the lowest binding free energy (ΔG, kcal/mol) was prioritized. Intermolecular interactions were analyzed using PyMOL 2.6 (Schrodinger, 2015) and LigPlot+ (Laskowski & Swindells, 2011) to provide structural context for the predicted mechanisms. Docking scores are summarized in Supplementary Table S2.

## 3 Results and discussion

### 3.1 Total phenolic/ flavonoid contents and bioactivities of *P. guajava* leaves

#### 3.1.1 Sample provenance and analytical system validation

**Fig. 1:**
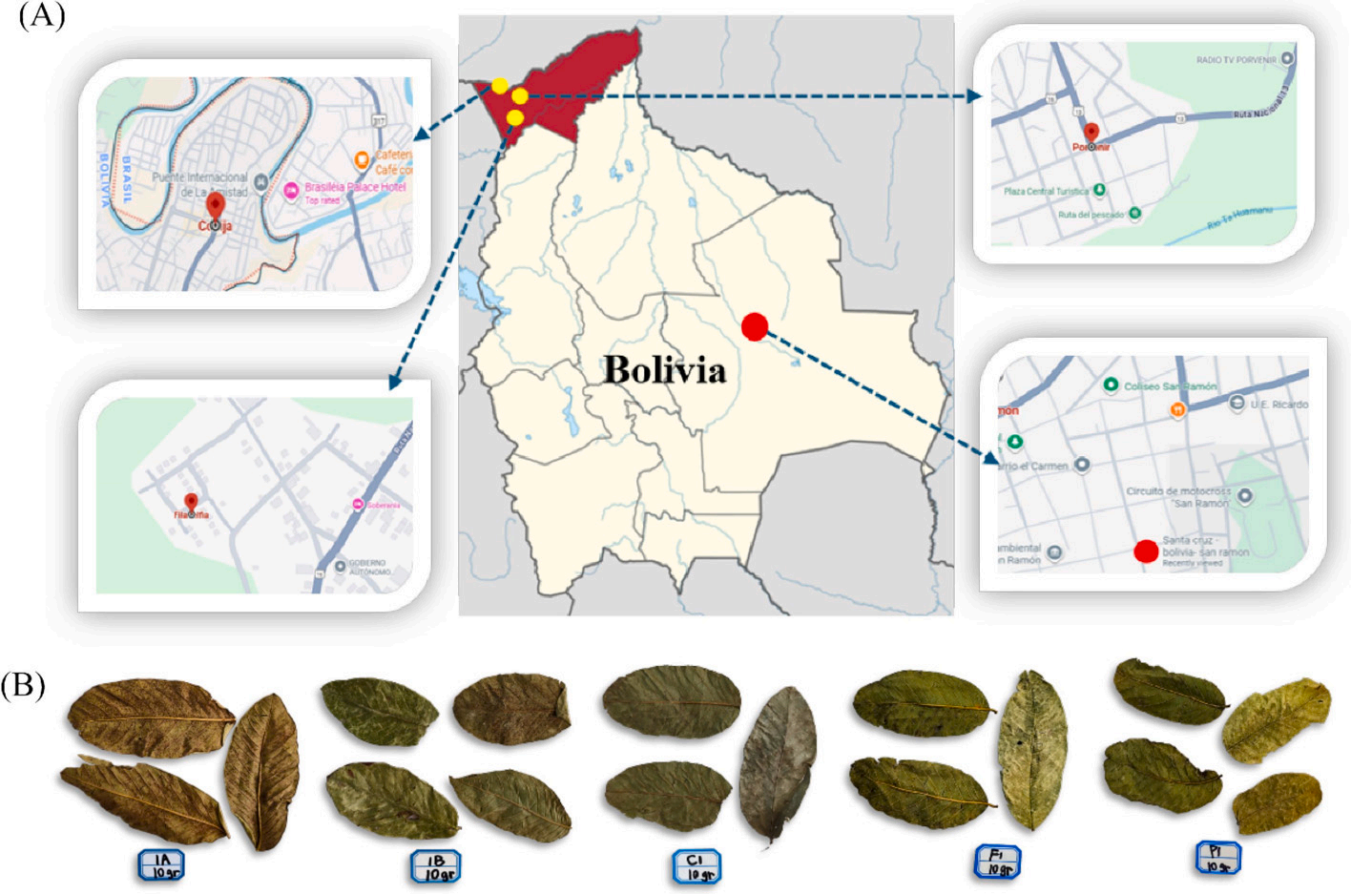
Geographical provenance and morphological features of *P. guajava* leaves. (A) Sampling locations across two contrasting eco-regions in Bolivia: A1–B1 (Tropical Lowland, Santa Cruz) and C1/F1/P1 (Amazon Rainforest, Pando). (B) Representative leaf photographs showcasing regional variations in pigmentation and texture.

Five wild *P. guajava* leaf populations were collected from two ecologically contrasting regions in Bolivia: the Tropical Lowland (A1 and B1, Santa Cruz) and the Amazon Rainforest (C1, F1, and P1, Pando) (Fig. 1A). Preliminary visual characterization revealed discernible regional morphological differences, with Amazonian accessions generally exhibiting deeper green pigmentation and a more coriaceous leaf texture compared to their Lowland counterparts (Fig. 1B). Widely targeted LC–MS/MS analysis annotated a total of 1230 phenolic-related metabolic features. Analytical stability was confirmed by the tight clustering of quality control (QC) samples in subsequent multivariate analysis, ensuring that the observed chemical divergence reflects intrinsic biological variation rather than instrumental drift.

#### 3.1.2 Variation in total phenolic and flavonoid contents

**Fig. 2:**
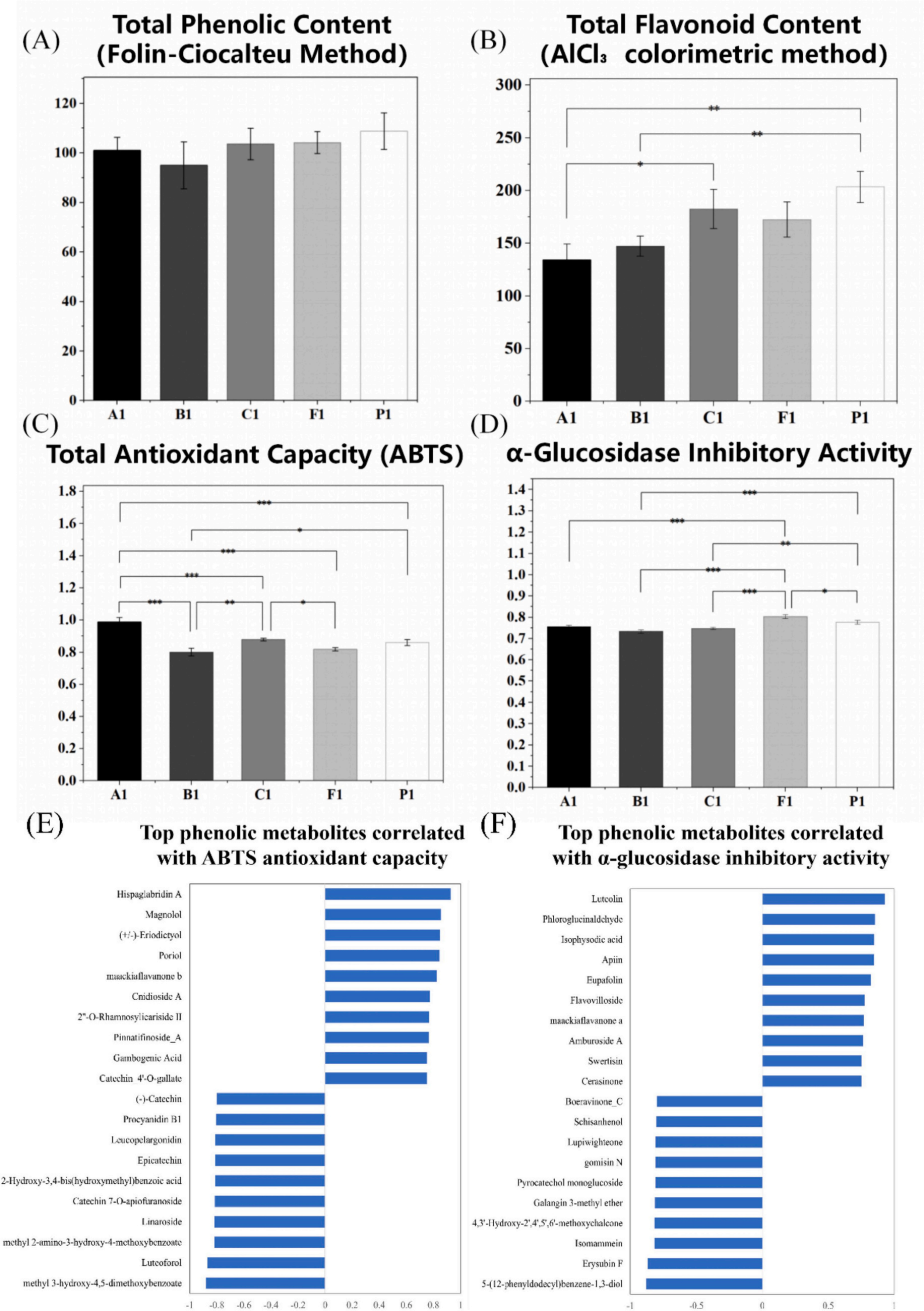
Phytochemical profiles, bioactivities, and metabolic correlations of *P. guajava* leaves. (A–D) TPC, TFC, ABTS scavenging capacity, and *α*-glucosidase inhibition (*n* = 3). (E–F) Top 20 metabolites correlated with ABTS and *α*-glucosidase inhibition (Spearman's ρ, *n* = 15). Statistical significance: *p < 0.05, **p < 0.01, ***p < 0.001 (ANOVA/Tukey) or BH-FDR adjusted.

The total phenolic content (TPC) across the five Bolivian sampling sites ranged from 94.99 to 108.85 mg GAE/g DW (Fig. 2A). One-way ANOVA revealed no significant differences among the sites (*p* > 0.05), indicating that the overall pool of reducing polyphenols in *P. guajava* leaves remains relatively stable at the species level under standardized extraction conditions (60% aqueous ethanol). These values align well with the ranges reported in previous studies for European-grown guava (86.12–113.34 mg/g DW) (Díaz-de-Cerio et al., 2016), confirming that high phenolic accumulation is a robust biological feature of *P. guajava* across diverse geographical environments.

However, because the Folin–Ciocalteu assay captures total reducing capacity and can be influenced by non-phenolic reductants, these bulk indices may mask underlying compositional shifts. In contrast, the total flavonoid content (TFC) differed markedly among sites (134.47–203.50 mg RE/g DW; Fig. 2B). Significant differences were observed (*p* < 0.01), particularly for the Amazonian site P1 (203.50 ± 14.31 mg RE/g DW), which substantially surpassed the tropical lowland sites A1 (134.47 ± 14.60 mg RE/g DW) and B1 (147.13 ± 9.42 mg RE/g DW). This decoupling between a stable TPC and a variable TFC suggests that regional environmental factors in Bolivia specifically fine-tune the partitioning of carbon precursors toward flavonoid-specific biosynthetic branches, rather than altering the primary flux into the general phenylpropanoid pathway.

The observed TFC, particularly the elevated levels in P1, is consistent with high-yield thresholds reported for wild germplasms. Notably, our raw values (134.47–203.50 mg RE/g DW) show excellent numerical coherence with the benchmark data, which reported a peak TFC of 198.64 ± 1.23 mg/g RE for *P. guajava* leaf extracts using an identical rutin-calibrated spectrophotometric system (Naseer et al., 2018). This direct alignment validates the enhanced flavonoid biosynthetic capacity of native Bolivian accessions under regional environmental pressures.

Because the spectrophotometric response of the aluminum chloride (AlCl₃) complexation assay is highly dependent on the microenvironment and the specific chemical structure of individual flavonoids (Pękal & Pyrzynska, 2014), no single colorimetric standard can accurately reflect the entire pool across different sub-families (Chang et al., 2002). Given that the flavonoid profile of *P. guajava* leaves is naturally dominated by quercetin glycosides (such as avicularin and guajaverin), utilizing a glycosylated standard like rutin provides a more structurally representative macro-index than a free aglycone standard such as quercetin, where the absence of C-3 glycosylation alters steric hindrance and chelation kinetics (Chang et al., 2002; Pękal & Pyrzynska, 2014). These colorimetric values must therefore be treated as standard-dependent relative metrics rather than interchangeable absolute values. To overcome the inherent limitations of macro-colorimetric screening, widely targeted LC-MS/MS analysis was subsequently performed (Section 3.2) to achieve chemically specific characterization of individual flavonoid monomers.

#### 3.1.3 Geographical variation in antioxidant and α-glucosidase inhibitory activities

Antioxidant capacity, measured by the ABTS assay, differed significantly among sites (*p* < 0.05), ranging from 0.8004 ± 0.0236 to 0.9880 ± 0.0278 mmol TE/g DW (Fig. 2C). Notably, site A1 showed the highest ABTS scavenging activity despite presenting an intermediate TPC and a comparatively low TFC. This discrepancy indicates that the antioxidant performance of *P. guajava* leaves cannot be inferred solely from bulk colorimetric indices. This pattern aligns with established structure-activity relationship (SAR) principles, where free radical scavenging capacity depends more on specific structural features, such as the relative abundance of free-form flavonoid aglycones versus sterically hindered glycosylated counterparts than on absolute phenolic abundance (Díaz-de-Cerio et al., 2017; Wang et al., 2018). The elevated antioxidant activity of A1 suggests a defensive metabolic adaptation to the high-irradiance and arid conditions of the tropical lowlands, where a higher relative abundance of aglycones may be required to mitigate photo-oxidative stress.

All extracts exhibited pronounced *α*-glucosidase inhibition at the tested concentration (200 μg/mL), with fractional inhibition values exceeding 0.73 (Fig. 2D). Acarbose (50 nM) was included as an internal positive control, yielding an inhibition fraction of 0.564 under identical assay conditions. The crude leaf extracts from native Bolivian accessions demonstrated strong inhibitory profiles, with the prominent Amazonian accession (F1) exhibiting a notable inhibition fraction of 0.804. This substantial bioactive capacity, achieved even in a non-purified state, underscores the bioprospecting potential of these wild germplasms for metabolic health applications. Such robust enzyme inhibition is typically driven by specific bioactive clusters, particularly flavones and related flavonoid aglycones, which effectively occupy the catalytic site of *α*-glucosidase. The maintenance of potent inhibition across sites with varying bulk TFC levels further supports the hypothesis that functional efficacy is governed by the specific profile of high-affinity inhibitors rather than total phenolic mass. This functional discrepancy effectively bridges the transition from non-specific colorimetric quantification to the metabolite-resolved characterization detailed in Section 3.2.

#### 3.1.4 Correlation between bulk indices and metabolite features

Pearson correlation analysis (*n* = 15) was performed to evaluate the associations between bulk phytochemical indices (TPC and TFC) and biological activities (Supplementary Data S1). TPC exhibited a negligible correlation with ABTS scavenging capacity (*r* = 0.076), while TFC showed a weak negative correlation (*r* = −0.364). For *α*-glucosidase inhibition, only moderate positive correlations were observed for both TPC (*r* = 0.431) and TFC (*r* = 0.371). The absence of strong linear associations indicates that bulk colorimetric indices are insufficient to explain the functional heterogeneity of Bolivian guava leaves, highlighting a clear decoupling between total phenolic abundance and biological efficacy. This pattern has been similarly observed in other polyphenol-rich genetic resources, where antioxidant performance is dictated by the prevalence of specific individual molecules rather than the total pool (Dzhanfezova et al., 2020).

This phenomenon aligns with the “Structure-Activity Predominance” theory (Rice-Evans et al., 1996), which asserts that polyphenol bioactivity is governed by specific structural motifs, such as the B-ring catechol group and the C2–C3 double bond, rather than absolute concentration. Consequently, a geographical site accumulating a higher proportion of high-potency molecules can exhibit enhanced bioactivity despite a relatively lower TPC. The stability of TPC alongside the marked inter-site variation in TFC supports a metabolic redirection hypothesis: under regional environmental pressures, plants may selectively divert carbon flux toward specific flavonoid branches within the broader phenylpropanoid pathway without necessarily expanding the total phenolic pool.

To identify the specific chemical drivers, a feature-level screening was conducted using Spearman rank correlations with Benjamini-Hochberg false discovery rate (BH-FDR) control. The ABTS-associated features revealed a distinct metabolic signature (Fig. 2E). Baseline monomeric flavan-3-ols, including (−)-catechin, epicatechin, and procyanidin B1, exhibited negative correlation trends with antioxidant capacity. In contrast, the top positive drivers were the flavanone (+/−)-eriodictyol and gallated derivatives such as catechin 4’-O-gallate. This pattern suggests that enhanced antioxidant performance in these wild ecotypes is likely driven by the downstream bioconversion of monomeric catechins into more complex gallated forms, or by the prevalence of chemotypes rich in ortho-diphenolic structures such as eriodictyol, which possess high single-electron transfer capacities (Pietta, 2000).

The correlation profile for *α*-glucosidase inhibition was distinctly orthogonal to the ABTS drivers, being primarily dominated by specific flavones (Fig. 2F). Luteolin emerged as the top positive correlate, followed by apiin and swertisin. Luteolin is increasingly recognized as a potent natural *α*-glucosidase inhibitors, with well-documented in vitro and in vivo efficacy driven by its high binding affinity to the enzyme's catalytic site (Xu et al., 2024). These distinct metabolic signatures demonstrate that the antioxidant and hypoglycemic potentials of *P. guajava* leaves are governed by discrete phytochemical subsets, providing a rigorous rationale for the comprehensive metabolomics analysis in Section 3.2.

### 3.2 Widely-targeted metabolomic profiling of *P. guajava* leaves

#### 3.2.1 Multivariate analysis reveals region-associated phenolic chemotype patterns

**Fig. 3:**
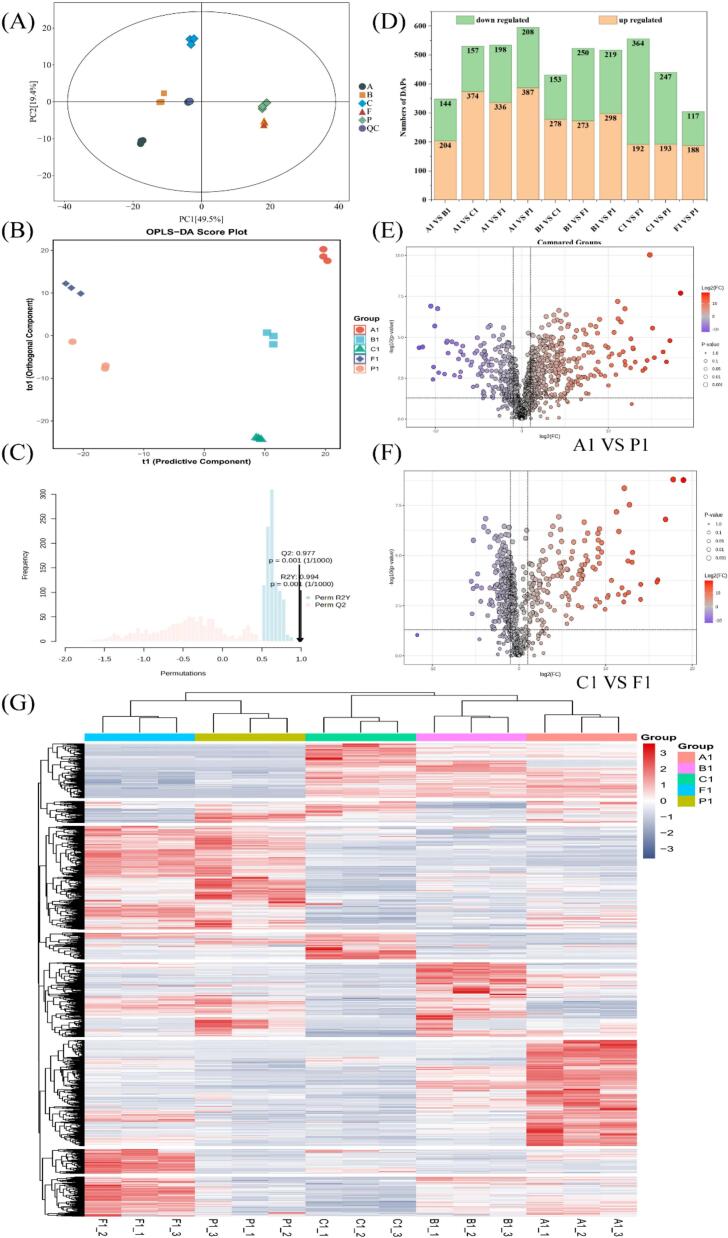
Multivariate and differential phenolic profiling of *P. guajava* leaves. (A) PCA score plot of annotated phenolics. (B—C) OPLS-DA score plot of identified phenolics (R^2^Y = 0.994, Q^2^ = 0.977) and 1000-iteration permutation test (*p* = 0.001). (D) Counts of differentially accumulated metabolites (DAMs) identified in pairwise comparisons between geographical sites. (E–F) Volcano plots for A1 vs. P1 and C1 vs. F1. (G) Hierarchical clustering analysis (HCA) heatmap of prioritized DAMs. DAMs were defined using a threshold of VIP > 1.0, BH-FDR < 0.05, and |log₂FC| ≥ 1.0.

Widely targeted LC–MS/MS analysis annotated a total of 1230 phenolic metabolites across the *P. guajava* leaf samples. A detailed parameter-by-parameter comparative matrix contrasting our analytical platform, metabolite coverage, and conceptual advances against representative benchmark literature is presented in Table S3 (Supplementary Materials). Analytical platform stability was confirmed by the tight clustering of QC samples and the minimal dispersion among biological replicates (Fig. 3A). Unsupervised PCA revealed clear origin-related spatial segregation (PC1: 49.5%; PC2: 19.4%), with Santa Cruz (A1/B1) and Amazonian (F1/P1) accessions clustering into distinct metabolic poles. This grouping indicates that the observed metabolic divergence reflects coordinated shifts across multiple phenolic subclasses rather than isolated compound-level changes. Hierarchical cluster analysis (HCA, Fig. 3G) further corroborated the transitional topology observed in the PCA projection. The dendrogram revealed a primary bifurcation: samples F1 and P1 formed a highly conservative sub-cluster representing the core Amazonian chemotype, whereas A1 and B1 tightly clustered to define the Lowland chemotype. Sample C1 occupied an intermediate hierarchical node, merging with the A1/B1 clade at a secondary level before joining the overarching F1/P1 cluster. Rather than representing a discrete anomaly, C1 acts as a transitional accession along the eco-geographical continuum, illustrating that metabolic divergence across Bolivian accessions follows coordinated spatial gradients rather than abrupt phenotypic boundaries.

The A1–B1 chemotype (Santa Cruz) was characterized by significantly elevated levels of aglycones and prenylated derivatives, such as apigenin, corylin, and silibinin. This biosynthetic signature, enriched in lipophilic scaffolds, represents a specialized adaptive response to the high solar radiation and drought-induced oxidative stress of the tropical lowlands. According to the adaptive significance theory of secondary metabolites (Wink, 2010), such compositional shifts reflect evolutionary mechanisms whereby plants prioritize the synthesis of lipophilic markers to provide enhanced membrane protection against high solar radiation and drought-induced oxidative stress. Notably, the prevalence of these free-form aglycones is functionally relevant, as O-glycosylation has been shown to generally reduce the in vitro antioxidant and anti-diabetic potencies of flavonoids (Xiao, 2017).

In contrast, the F1–P1 cluster exhibited a pronounced enrichment in glycosylated polyphenols, including limocitrol 3-glucoside and myricitrin. This shift suggests a biosynthetic flux prioritized toward glycosylation, a strategy frequently employed by plants in high-humidity Amazonian environments to enhance the water solubility, stability, and vacuolar storage of phenolics under biotic pressures (Xiao, 2017). While these glycoside-bound forms may serve as a stabilized metabolic reservoir, their accumulation results in a distinct functional profile compared to the aglycone-rich counterparts. Overall, these results demonstrate that Bolivian *P. guajava* leaves have evolved divergent phenolic chemotypes, and that this metabolic segregation, dictated by regional agro-ecological conditions, serves as the primary chemical driver for the functional variations observed across the different geographical sites.

#### 3.2.2 Identification of differential phenolic metabolites across origins

While PCA and HCA provided an unsupervised overview, supervised OPLS-DA was employed to prioritize features contributing to regional segregation (Fig. 3B). The model demonstrated high goodness-of-fit (R^2^Y = 0.994) and predictive capability (Q^2^ = 0.977), rigorously validated by a 1000-iteration permutation test (*p* = 0.001; Fig. 3C). Differentially accumulated metabolites (DAMs) were defined using a multidimensional threshold: Variable Importance in Projection (VIP) > 1.0, a two-sided *t*-test with Benjamini–Hochberg FDR correction (*p*_adj_ < 0.05), and a fold change |log₂FC| ≥ 1.0. This rigorous filtering identified a substantial number of phenolic DAMs, with the magnitude of metabolic divergence reflecting the geographical distance and environmental contrast between site pairs (Fig. 3D). Specifically, the comparison between the semi-arid Santa Cruz (A1) and the humid Amazonian Pando (P1) regions yielded the largest differential set (595 metabolites, Fig. 3E), followed by the Amazonian intra-regional comparison of C1 vs. F1 with 556 DAMs (Fig. 3F). This profound divergence, annotating nearly half of the total phenolic features as DAMs, underscores the influence of distinct agro-ecological zones on the leaf's phenolic architecture. The complete pairwise outputs are provided in Supplementary Figs. S2–S3.

To refine the candidate set for subsequent in silico validation, DAMs were further prioritized based on MS/MS-supported annotation confidence, robust signal intensity, and consistent differential trends across geographical comparisons. This multi-step prioritization ensured that only the most representative “marker metabolites” were selected for network pharmacology and molecular docking, in alignment with established metabolomic workflows for identifying chemical markers that define distinct plant ecotypes (Chen et al., 2013).

#### 3.2.3 Chemical signatures and eco-physiological adaptations underlying origin-dependent profiles

The distribution of phenolic DAMs across multiple subclasses reflects a coordinated metabolic response to regional agro-ecological constraints. A defining feature of these geographical signatures was the sharp divergence in flavonoid conjugation patterns. Samples from the Santa Cruz region (A1–B1), characterized by distinct seasonal fluctuations and lower annual precipitation (≈ 1200 mm), exhibited a specialized enrichment in lipophilic aglycones and prenylated flavonoids, such as silibinin and longistylin A.

From an ecological perspective, this accumulation aligns with the defensive strategy of plants in arid habitats. Free flavonoid aglycones typically occur externally on leaf surfaces or within secretory structures and often correlate with the production of other lipophilic secondary metabolites (Wollenweber, 1982). This spatial localization serves as a robust chemical barrier to mitigate UV-induced damage and excessive transpiration in semi-arid environments (Tomás-Barberán & Wollenweber, 1990).

In stark contrast, accessions from the high-humidity Amazonian basin (F1 and P1) demonstrated a significant metabolic shift toward glycosylated conjugates. This “glycosylation signature” represents a strategic adaptation where plants utilize glycosylation to enhance the aqueous solubility and chemical stability of phenolics for efficient vacuolar sequestration (Harborne, 1993). Beyond flavonoids, the site-specific variation follows the stress-induced phenylpropanoid metabolism model, where metabolic flux is selectively diverted toward specific protective branches while maintaining a baseline total phenolic output (Dixon & Paiva, 1995). This coordinated reorientation of secondary metabolism provides a rigorous chemical foundation for the distinct biological activities reported in Section 3.1, justifying the prioritization of these origin-specific markers for subsequent network pharmacology and molecular docking analyses.

#### 3.2.4 Target-specific verification of geographical markers and structural divergence

Flavonoids function as versatile ‘molecular shields’ in plant–environment interactions, mediating non-enzymatic antioxidant responses to mitigate oxidative damage induced by abiotic stressors (Patil et al., 2024). The synthesis of these polyphenols is fundamentally driven by the transcriptional activation of the phenylpropanoid pathway (Sharma et al., 2019), a process specifically amplified in *P. guajava* by drought stress to reinforce systemic stress retaliation (Usman et al., 2022).

**Fig. 4:**
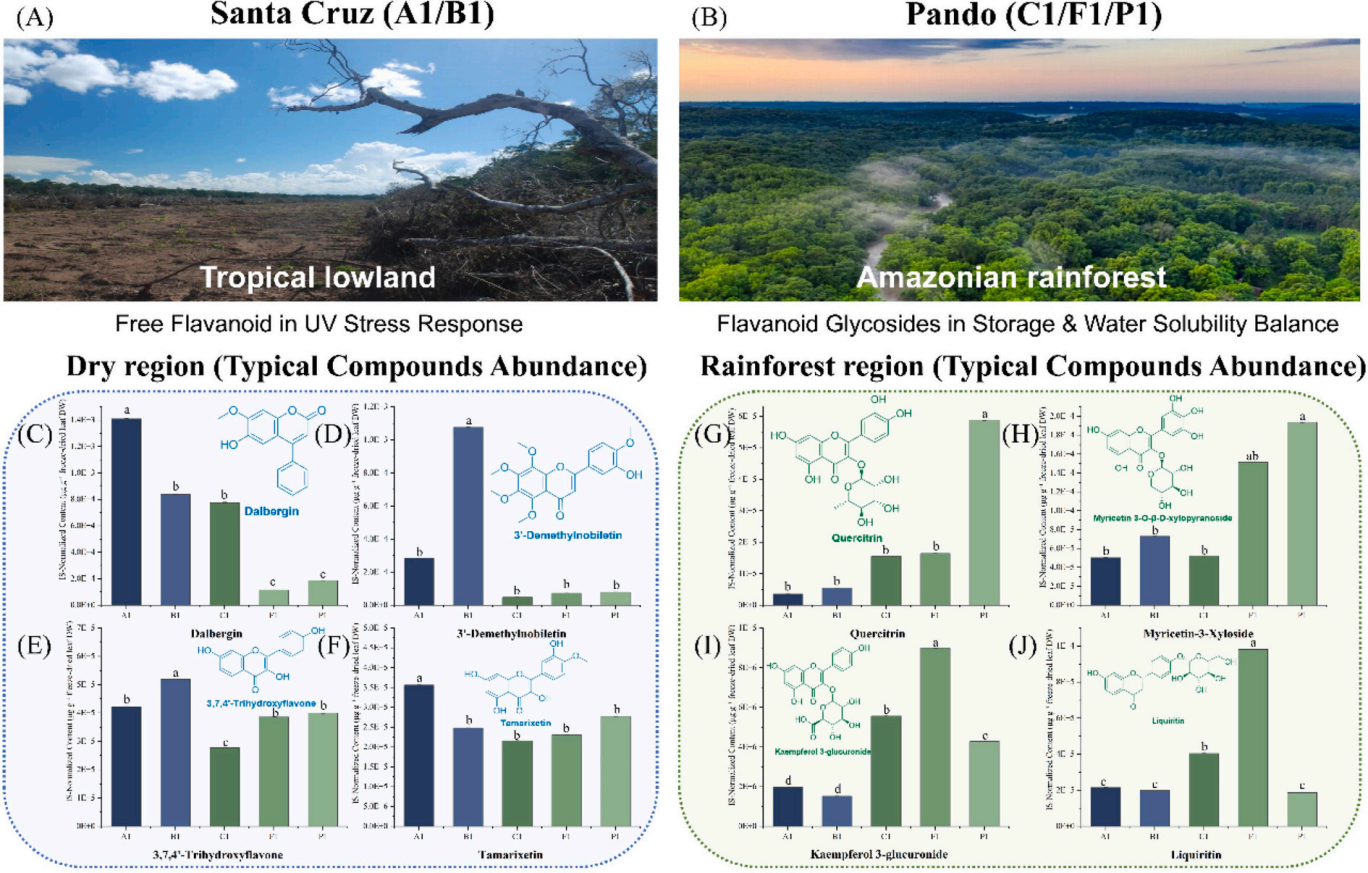
Ecological driving forces and metabolic divergence of prioritized markers in *P. guajava* leaves. (A–B) Representative agro-ecological landscapes of the semi-arid Santa Cruz and Amazonian Pando regions. (C–J) Relative abundance of eight geographical markers categorized by structural attributes: Group I (C—F) represents lipophilic aglycones and neoflavonoids (e.g., Dalbergin) specifically enriched under high-irradiance SC conditions; Group II (G–J) comprises hydrophilic flavonoid glycosides (e.g., Quercitrin) predominantly sequestered in the Pando region. Data are presented as mean ± SD (n = 3). Different lowercase letters (a–c) denote significant differences among regions (*p* < 0.05, one-way ANOVA followed by Tukey's test).

Consistent with these defensive strategies, the metabolic profile of the semi-arid Santa Cruz region reveals a specialized accumulation of free aglycones (Fig. 4). This aglycone-rich signature, representative of the characteristic *P. guajava* defensive scaffolds such as dihydrochalcones and stilbenes (Rojas-Garbanzo et al., 2017), reflects a strategic metabolic shift toward extravacuolar localization. Due to their high lipophilicity, these non-glycosylated compounds are preferentially deposited within the leaf epidermis and hypodermis, forming a primary chemical barrier (Mierziak et al., 2014). This localization facilitates the interception of deleterious UV-B radiation at the pre-cytoplasmic level, effectively shielding sensitive intracellular organelles (Tattini et al., 2004). Beyond structural screening, this divergence yields ‘effective antioxidants’ capable of scavenging ROS directly at their sites of generation (Agati & Tattini, 2010). Furthermore, the integration of these non-polar compounds into the cuticular matrix bolsters the leaf's hydrophobic properties to mitigate transpiration-driven water loss (Tattini et al., 2004), while maintaining signaling integrity under compounded environmental pressures (Brunetti et al., 2013; Qaderi et al., 2023).

Conversely, samples from the Amazonian Pando region exhibited a distinct metabolic footprint characterized by the enrichment of hydrophilic flavonoid glycosides, such as quercitrin and myricetin-3-xyloside (Group II, Fig. 4G–J). Given that flavonoid aglycones are inherently unstable and potentially cytotoxic, glycosylation serves as a requisite stabilization mechanism for safe sequestration (Ren et al., 2024; Vogt & Jones, 2000). This ‘glycosylation signature’ represents an energy-efficient storage strategy to maintain metabolic homeostasis in the humid rainforest understory (Zhang et al., 2022). Under the “binary chemical defense” model, these stored glycosides serve as inert precursors that, upon tissue damage or fungal infection, are rapidly hydrolyzed by endogenous *β*-glucosidases to deploy active defense moieties against invading pathogens (Dixon, 2001; Morant et al., 2008). Notably, quercitrin has been identified as a high-value scaffold with significant potential in managing metabolic disorders (Ren et al., 2024). Building on these structural variations, we further explored the molecular interactions of these prioritized markers within the AGE-RAGE signaling pathway to evaluate their site-specific bioactivity.

### 3.3 Identification and prioritization of phenolic candidates

To facilitate downstream in silico mechanism exploration, the metabolic matrix of 1230 annotated phenolics was subjected to a tiered prioritization workflow (Supplementary Methods). Pairwise comparisons across the five sampling sites revealed 753 unique DAMs, representing 61.2% of the total phenolic profiles. This substantial proportion of differential features underscores the decisive influence of geographical origin on the chemical architecture of the leaves.

To ensure biological relevance and minimize signal redundancy for subsequent systems pharmacology, the DAM pool was systematically distilled. From an initial subset of 365 Tier-1 and Tier-2 metabolites, a high-confidence cohort of 147 phenolic candidates was curated (Supplementary Data S2). In strict adherence to the Metabolomics Standards Initiative (MSI) guidelines (Sumner et al., 2007), these candidates were classified as Level 2 (putatively annotated compounds) based on the high-fidelity matching of their MS/MS fragmentation patterns against public and in-house spectral libraries. Prioritization was governed by three stringent criteria: (i) robust structural elucidation supported by high-quality secondary mass spectrometry; (ii) a substantial biological effect size (|log₂FC| ≥ 2.0); and (iii) the availability of verifiable database identifiers (e.g., PubChem CID, SMILES) required for high-fidelity target prediction.

This refined set of 147 markers represents the “core bioactive potential” of Bolivian *P. guajava* leaves and served as the foundational input for subsequent systems pharmacology and molecular docking analyses, bridging the gap between site-specific metabolic signatures and their underlying mechanistic hypotheses.

### 3.4 Network pharmacology analysis

#### 3.4.1 Identification of intersecting targets

**Fig. 5:**
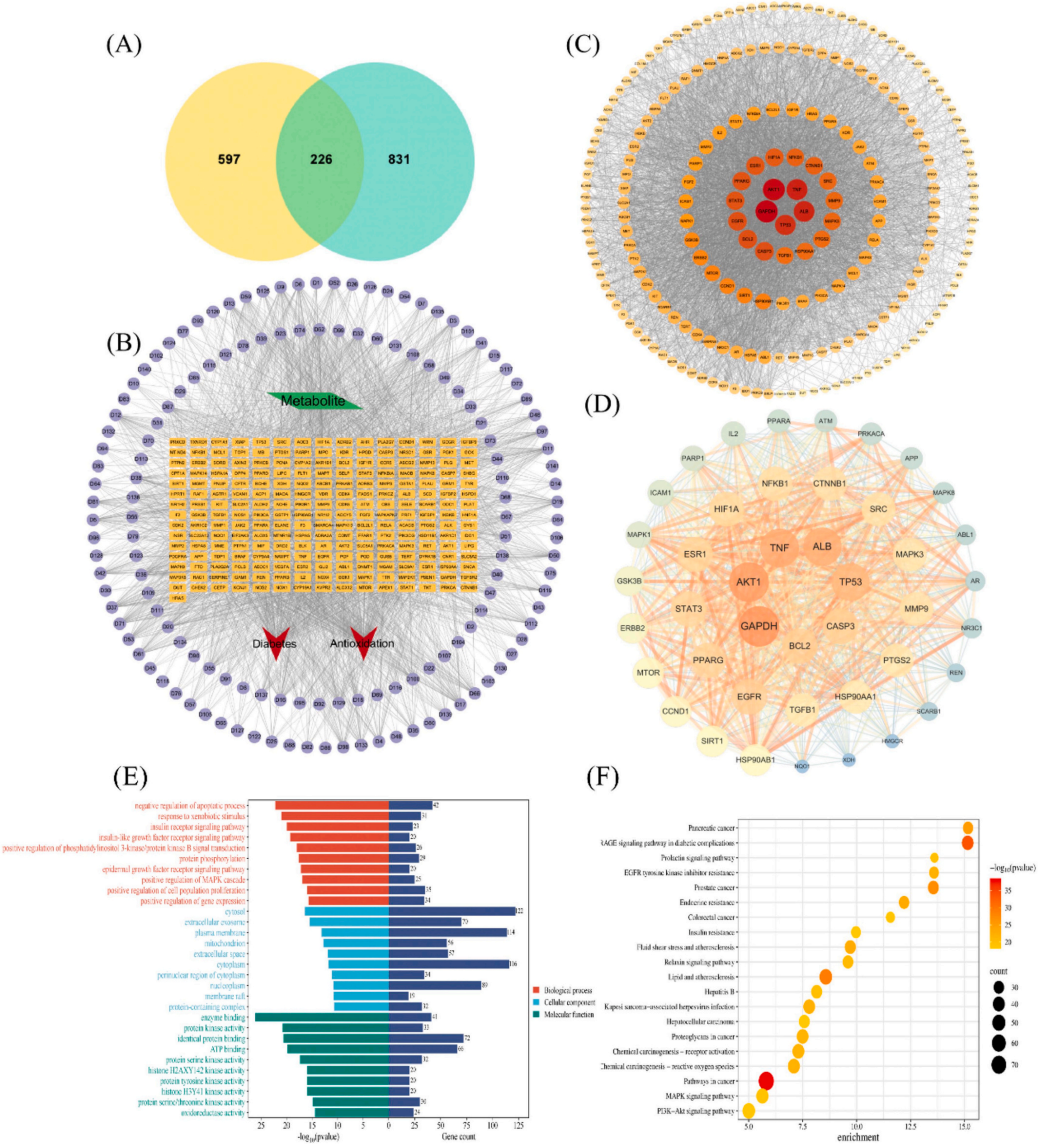
Network pharmacology integration linking origin-specific markers to diabetes and oxidative stress. (A) Venn diagram illustrating the intersection between predicted metabolite targets and disease-associated protein sets. (B) Metabolite–target–disease network for the 226 shared targets. (C) Protein-protein interaction (PPI) network derived from the STRING database. (D) Core PPI sub-network prioritized by degree, betweenness, and closeness centrality. (E) Gene Ontology (GO) enrichment analysis highlighting biological processes, cellular components, and molecular functions. (F) KEGG pathway enrichment analysis of the intersecting targets.

Based on the 147 prioritized candidates, a network pharmacology workflow was implemented to explore the multi-target mechanisms underlying the observed hypoglycemic and antioxidant effects. Computational target prediction yielded 535 putative human protein targets, which were subsequently intersected with 1057 high-relevance disease targets associated with diabetes and oxidative stress (Supplementary Data S3–S4). As shown in Fig. 5A, the resulting 226 common targets underscore the multi-component, multi-target synergistic potential inherent in the *P. guajava* phenolic matrix, reflecting the hallmark “poly-pharmacology” paradigm for complex metabolic disorders (Hopkins, 2008).

#### 3.4.2 Functional enrichment underscores the AGE–RAGE and PI3K/Akt axes

To elucidate the systematic regulation orchestrated by these targets, KEGG enrichment analysis identified the AGE–RAGE signaling pathway (BH-FDR = 1.89 × 10^−34^; enrichment factor = 15.15; Fig. 5F) as the most statistically prominent module. The AGE–RAGE axis is a pivotal driver of diabetic progression, where the accumulation of advanced glycation end-products (AGEs) triggers RAGE-mediated oxidative cascades and chronic inflammation (Yamagishi, 2019). This computational prediction aligns with established biochemical evidence that *P. guajava* leaf phenolics, specifically their core scaffolds like quercetin and gallic acid, act as potent antiglycation agents that effectively block AGE formation (Wu et al., 2009). Consistent with the KEGG findings, GO enrichment prioritized biological processes related to the positive regulation of PI3K/Akt signaling (BH-FDR = 1.00 × 10^−18^; Fig. 5E). The PI3K/Akt axis is a critical node for both glucose disposal and redox homeostasis; its biological relevance is substantiated by previous in vivo findings demonstrating that *P. guajava* leaf extracts can attenuate insulin resistance in diabetic models via activation of this signaling pathway (Yang et al., 2020). Together, these systems-level insights transition our study from in vitro observations to a pathway-oriented hypothesis: origin-dependent phenolic profiles may exert anti-diabetic effects by suppressing RAGE-mediated oxidative damage while restoring PI3K/Akt-dependent insulin sensitivity.

#### 3.4.3 PPI network construction and core target identification

To resolve the functional architecture of the 226 common targets, a PPI network was constructed using the STRING database and visualized in Cytoscape (Fig. 5C). Comprehensive topological metrics, including degree, betweenness centrality, and closeness centrality were calculated to pinpoint the most influential nodes within the interactome (Supplementary Data S5).

Initial topological screening identified several high-centrality nodes, notably AKT1, TNF, GAPDH, ALB, and TP53. However, rather than relying solely on connectivity, which can be biased by ubiquitous proteins (Goh et al., 2007), we implemented a biologically driven prioritization strategy by intersecting the PPI topology with the AGE–RAGE-centric enrichment results (Section 3.4.2). While GAPDH, ALB, and TP53 exhibited exceptionally high degree values, they typically function as ubiquitous “housekeeping” proteins and lack functional specificity regarding *P. guajava*'s anti-diabetic effects, these nodes were therefore excluded from further mechanistic validation.

In contrast, a refined panel consisting of AKT1 and TNF, AGER (RAGE), RELA (NF-*κ*B p65), and MAPK14 (p38*α*) was prioritized. This selection spans the entire pathological axis of diabetic complications: from surface receptor activation (AGER) to downstream kinase-mediated signal transduction (MAPK14 and AKT1) and transcriptional regulation of inflammatory output (RELA and TNF) (Bierhaus et al., 2005). The functional interconnectedness of this panel is substantiated by pharmacological evidence that RAGE engagement activates the PI3K/Akt, MAPK, and NF-*κ*B signaling cascades, thereby driving oxidative stress and chronic tissue inflammation in diabetic environments (Sanajou et al., 2018). This framework provides a biologically grounded basis for evaluating the site-specific bioactivity of Bolivian guava phenolics via molecular docking.

#### 3.4.4 Target prioritization for molecular docking

Integrating KEGG enrichment with PPI topology, AGER, RELA, TNF, AKT1, and MAPK14 were prioritized as the core docking panel. These targets occupy complementary functional niches across the AGE–RAGE signaling cascade, bridging surface receptor activation with downstream kinase signaling. This selection enables a systematic evaluation of whether the prioritized Bolivian guava phenolics exert a holistic pathway disruption rather than an isolated effect on a single network hub.

### 3.5 Molecular docking provides structural insights into potential multi-target interactions

To assess the binding potential of identified markers with core signaling nodes, systematic molecular docking was performed. Thermodynamic stability was assessed against established computational thresholds: binding energies below −5.0 kcal/mol indicate favorable interactions (Hsin et al., 2014), while scores below −7.0 kcal/mol suggest high-affinity leads (Shityakov & Förster, 2014). In this study, the prioritized markers exhibited predicted multi-target binding energies ranging from −7.3 to −10.1 kcal/mol, suggesting stable structural complementarity against the key regulatory proteins of the AGE–RAGE and inflammatory pathways (Table S2).

**Fig. 6:**
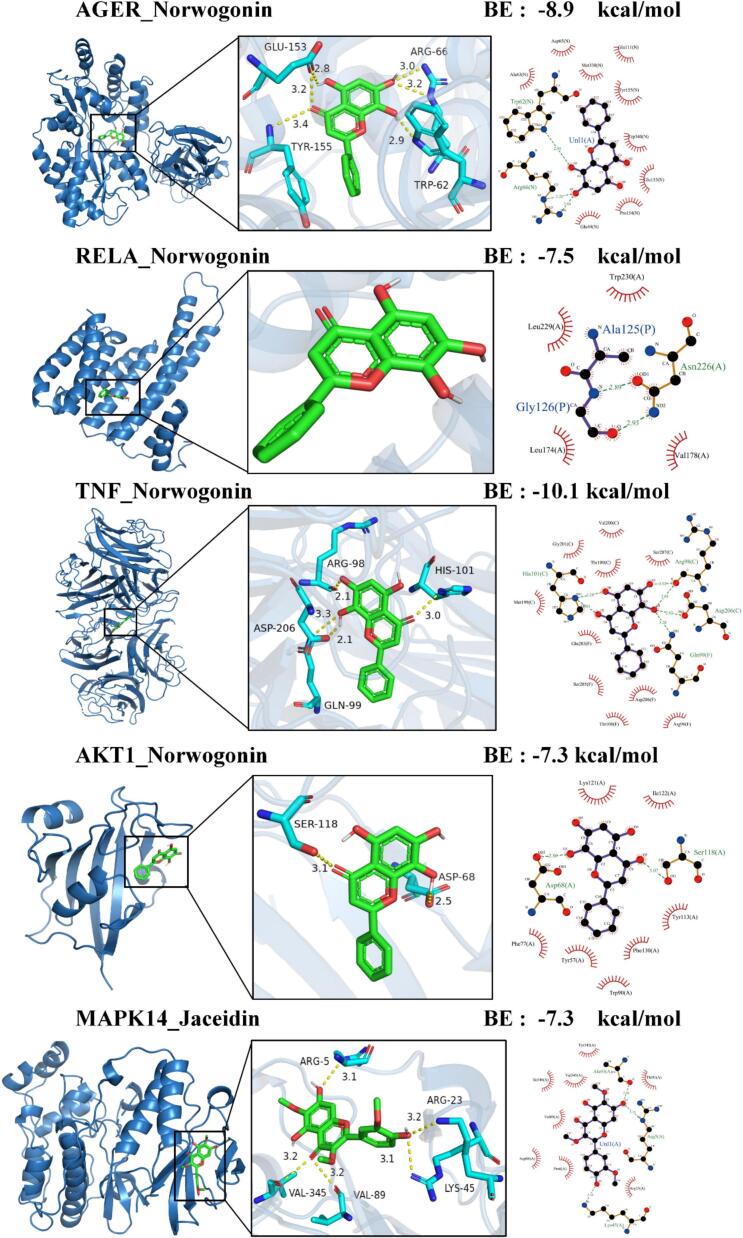
Molecular docking simulations of Norwogonin and Jaceidin with five key targets (AGER, RELA, TNF, AKT1, and MAPK14). Profiles illustrate the lowest-energy conformations, local binding geometries, and corresponding 2D interaction maps. Binding scores are indicated in kcal/mol.

As shown in Fig. 6, norwogonin was predicted to possess a potent binding affinity for TNF (−10.1 kcal/mol), stabilized by a predicted hydrogen-bonding network with HIS101, ARG98, and ASP206. Its predicted interactions with AGER (−8.9 kcal/mol; ARG66, TRP62) and AKT1 (−7.3 kcal/mol; ASP68, SER118) further suggest its potential capacity to modulate the inflammatory cascade. These predicted affinities offer a plausible structural rationale for the significant downregulation of TNF–*α* and associated inflammatory markers reported in related botanical extract studies (Wang et al., 2025). Notably, the theoretical binding modes observed for Norwogonin are consistent with recent pharmacological data, including enzymatic inhibition and SPR data (Cao et al., 2025).

Jaceidin was predicted to exhibit strong binding affinities for MAPK14 (−7.3 kcal/mol) and RELA (−7.5 kcal/mol), theoretically anchored by H-bonds with ARG5/LYS45 and a hydrophobic network involving TRP130/LEU229/LEU174/VAL178, respectively. The 3,6,3′-trimethoxy pattern of Jaceidin aligns with established SAR principles, where C-3 *O*-methylation enhances inhibitory activity against inflammatory mediators while minimizing cytotoxicity (Matsuda et al., 2003). This theoretical binding potential is further supported by literature evidence from its structural analog, Jaceosidin, which suppresses MAPK14 phosphorylation and NF-*κ*B activation (Nam et al., 2013), and targets PKM2 to regulate immunometabolic reprogramming (Dai et al., 2025).

In summary, these findings represent the first identification of Norwogonin and Jaceidin as potential multi-target constituents in Bolivian guava. By integrating these computational binding affinities with metabolomic data, we propose that the prevalence of specific aglycones, rather than glycosylated derivatives, may provide a favorable molecular basis for modulating metabolic stressors associated with the AGE–RAGE axis. It is important to emphasize that all molecular docking findings are computational in silico predictions; future studies should prioritize bio-guided isolation and rigorous in vitro and in vivo validation to confirm these mechanistic hypotheses.

### 3.6 Integrated interpretation: From eco-physiological adaptation to functional quality divergence

This study establishes a multi-dimensional bridge connecting the regional origins of *P. guajava* in Bolivia with their specialized molecular mechanisms of action. The stark divergence between the Amazonian (Pando) and Tropical Lowland (Santa Cruz) chemotypes underscores a coordinated restructuring of the phenolic scaffold, likely driven by long-term eco-geographical stratification.

The prevalence of glycosylated flavonoids in the Pando region suggests a metabolic adaptation to high-humidity, high-precipitation rainforest environments. In these conditions, glycosylation serves as a requisite stabilization mechanism for vacuolar sequestration, enhancing the storage stability of antioxidants against fungal pathogens and biotic stressors (Zhang et al., 2022). This eco-physiological differentiation suggests that Amazonian guava leaves inherently possess a ‘glycoside-optimized’ profile, which may influence their extraction efficiency and sensory properties in functional tea formulations. Under the “binary chemical defense” model, these stored glycosides act as inert precursors that can be rapidly bio-activated upon tissue damage. Conversely, the enrichment of lipophilic aglycones and prenylated phenolics (e.g., norwogonin) in the seasonally dry Santa Cruz region reflects a specialized response to increased UV-B radiation and temperature fluctuations. Due to their high lipophilicity, these non-glycosylated compounds are preferentially deposited within the leaf epidermis and cuticular matrix, prioritizing extravacuolar chemical shielding and direct, non-enzymatic ROS scavenging at the site of generation (Tattini et al., 2004).

Crucially, this origin-dependent metabolic architecture represents a functional optimization rather than a merely quantitative shift. Our in silico findings reveal that the transition from a ‘glycoside-dominant’ (Pando) to an ‘aglycone-rich’ (Santa Cruz) chemotype results in distinct predicted interaction patterns with regulatory nodes of the AGE–RAGE and PI3K/Akt axes. While Pando accessions may provide receptor-level protection through polar interactions (e.g., myricitrin–RAGE), the Santa Cruz accessions may offer advantages in modulating intracellular signaling via deep-pocket binding of potent aglycones (e.g., norwogonin–AKT1). This specificity provides a computational basis for targeted dietary interventions. Consequently, chemotype ‘superiority’ is not an absolute metric but is strictly assay- and application-dependent: the Amazonian chemotype offers notable glycoside yield and surface enzymatic inhibition, whereas the Lowland chemotype may provide optimized intracellular target binding and direct antioxidant efficiency.

Together, these findings demonstrate that the geographical origin of guava leaves serves as a reliable biological “proxy” for specific chemical signatures. By integrating high-resolution metabolomics with network pharmacology, we have transitioned from traditional bulk-index evaluation to a chemotype-oriented quality standard. This framework clarifies why traditional TPC assays fail to predict biological efficacy and provides a data-driven model for the precision sourcing and standardized manufacturing of high-quality botanical ingredients.

## 4 Conclusion

This study establishes that geographical origin is the primary determinant governing the phenolic chemotype and functional specificity of Bolivian *P. guajava* leaves. Environmental divergence between Amazonian and Tropical Lowland habitats drives a metabolic trade-off between glycosylated and aglycone-type flavonoids. Specifically, the Lowland (Santa Cruz) accessions, enriched in lipophilic aglycones, exhibit enhanced ABTS radical-scavenging capacity, whereas the Amazonian accessions prioritize glycosylated flavonoids that drive pronounced *α*-glucosidase inhibition.

Crucially, the identified bioactive markers (such as norwogonin) and their predicted modulation of the AGE–RAGE and PI3K/Akt axes represent theoretical in silico predictions. Consequently, future studies should prioritize the bio-guided isolation, rigorous in vitro and in vivo mechanistic validations, and processing stability evaluations. Overall, these findings provide an origin-aware framework for the targeted sourcing and high-value utilization of guava leaf resources in functional foods.

## CRediT authorship contribution statement

**Xin-Yu Tao:** Writing – original draft, Visualization, Methodology, Investigation, Data curation, Conceptualization. **Zhou-Wei Wu:** Writing – review & editing, Formal analysis, Conceptualization. **Xiao-Cui Liu:** Writing – review & editing, Validation, Investigation. **Chen-Xi Quan:** Writing – review & editing, Validation, Investigation. **Marco Antonio Cabero:** Writing – review & editing, Investigation, Funding acquisition. **Ming-Hua Qiu:** Supervision, Project administration, Funding acquisition.

## Funding

This work was supported by the Beijing Natural Science Foundation (International Scientist “HuiZhi” Program, No. IS24049), Major Special Programe of science and technology of Yunnan (202402AA310032), as well as the Cooperation Project with China Biodiversity Conservation and Green Development Foundation (CBCGDF, 2025).

## Declaration of competing interest

The authors declare that they have no known competing financial interests or personal relationships that could have appeared to influence the work reported in this paper.

## Data Availability

The data supporting the findings of this study are publicly available in Mendeley Data at https://doi.org/10.17632/dnnvkddzdg.1.
