# Supplementary material for: Integrated metabolomics and network pharmacology to decipher habitat-driven metabolic diversity and α-glucosidase inhibitory mechanisms in guava leaves
Source: Food Chem X. 2026 Aug 3;38:104274. doi: 10.1016/j.fochx.2026.104274 (PMC13476485; doi:10.1016/j.fochx.2026.104274)
Supplement: Supplementary file 1 [file mmc1.docx]

**Supplementary Material**

**Integrated metabolomics and network pharmacology to decipher habitat-driven metabolic diversity and *α*-glucosidase inhibitory mechanisms in guava leaves.**

Xin-Yu Tao^a,b^, Zhou-Wei Wu^a,b^, Xiao-Cui Liu^a,b^, Chen-Xi Quan^a,b^, Marco Antonio Cabero^c,d^, Ming-Hua Qiu^a,b,*^

*^a^* State Key Laboratory of Phytochemistry and Natural Medicines, Kunming Institute of Botany, Chinese Academy of Sciences, Kunming 650201, Yunnan, China

*^b^* University of Chinese Academy of Sciences, Beijing 100049, China

*^c^* National Museum of Natural History, La Paz, Bolivia

*^d^* Andean Road Countries for Science and Technology (ARCST), La Paz, Bolivia.

*Corresponding author: Ming-Hua Qiu

**Supplementary Methods**

1. **Tiered Prioritization of Differentially Accumulated Metabolites (DAMs) for Downstream Interpretation**

To evaluate the biological importance of features identified as significant across the 10 pairwise comparisons by OPLS-DA and univariate statistics, a tiered prioritization system (Tier 1–3) was implemented independently of MS/MS annotation confidence to support downstream interpretation. DAMs were defined as VIP > 1.0, BH-FDR < 0.05, and |log₂FC| ≥ 1.0. Within a given comparison, DAMs with a large effect size (|log₂FC| ≥ 2.0) were additionally designated as “core” DAMs. For each metabolite, three cross-comparison summary statistics were calculated: (i) the number of comparisons in which it was statistically significant, (ii) whether it was designated as a core DAM in at least one comparison; and (iii) the maximum VIP and maximum |log₂FC| observed across all comparisons.

**Tier 1** (broad-spectrum core markers) required significance in ≥ 3 comparisons, core designation in ≥ 1 comparison, and max (VIP) ≥ 1.5. **Tier 2** (high-impact, comparison-specific markers) included DAMs significant in 1–2 comparisons that additionally met either max (|log₂FC|) > 3.0 or max (VIP) ≥ 2.0. **Tier 3** (supportive markers) comprised metabolites that were core in ≥ 1 comparison but did not satisfy Tier 1 or Tier 2 criteria, or those with max (VIP) ≥ 1.5 that were not statistically significant in any comparison. These tiers represent statistical prioritization only and are entirely independent of MS/MS identification confidence, as described in the main Materials and Methods (Section 2.6).

**Supplementary Data Descriptions (Available via Mendeley Data)**

The following large datasets (Supplementary Data S1–S6) are deposited and publicly available in Mendeley Data (DOI: 10.17632/dnnvkddzdg.1).

**Supplementary Data S1.** Pearson and Spearman rank correlation coefficients between bulk phytochemical indices (TPC, TFC) and biological activities (ABTS, AGI) across all 15 samples, with Benjamini–Hochberg FDR-adjusted p-values.

**Supplementary Data S2.** Complete list of 147 high-confidence phenolic candidates selected from the Tier 1–2 DAM pool, including PubChem CID, SMILES, MS/MS annotation confidence level (MSI Level 2), and prioritization tier assignment.

**Supplementary Data S3.** Disease-related target set for diabetes and oxidative stress retrieved from GeneCards (relevance score ≥ 5) and OMIM, and the 226 overlapping targets used for downstream network pharmacology analyses.

**Supplementary Data S4.** Predicted compound–target pairs from SwissTargetPrediction (Homo sapiens setting) for the 147 prioritized phenolic candidates, retaining only targets with non-zero probability.

**Supplementary Data S5.** Protein–protein interaction (PPI) network topology metrics (degree, betweenness centrality, closeness centrality) for the 226 common targets, with selected core nodes annotated.

**Supplementary Data S6.** Full Gene Ontology (GO) and KEGG pathway enrichment outputs for the 226 common targets, filtered at Benjamini–Hochberg adjusted p < 0.05.

**Fig. S1.** Multivariate patterns and overlap of differentially accumulated metabolites among guava leaf samples from five Bolivian origins. (A) PCA score plot of the processed intensity matrix (n = 15; QC samples included). (B) Venn diagram of differentially accumulated metabolites (DAMs) from A1-referenced pairwise comparisons (A1 vs B1/C1/F1/P1), defined as VIP > 1.0, BH-FDR < 0.05, and |log₂FC| ≥ 1.0. (C) Heatmap with hierarchical clustering of retained metabolite features across individual replicates (log-transformed, auto-scaled; row z-scores), generated in MetaboAnalyst 6.0 (Euclidean distance, Ward's linkage).

**Fig. S2.** Volcano plots of differentially accumulated phenolic metabolites in representative pairwise comparisons. (A) A1 vs B1, (B) A1 vs C1, (C) A1 vs F1, and (D) B1 vs C1. DAMs were defined as VIP > 1.0, BH-FDR < 0.05, and |log₂FC| ≥ 1.0. Positive log_2_FC indicates higher abundance in the first-listed origin.

**Fig. S3.** Volcano plots for additional representative pairwise comparisons of phenolic metabolites. (A) B1 vs F1, (B) C1 vs P1, (C) B1 vs P1, and (D) F1 vs P1. DAMs were defined as VIP > 1.0, BH-FDR < 0.05, and |log₂FC| ≥ 1. Positive log_2_FC indicates higher abundance in the first-listed origin.

**Table S1.** Pearson correlation coefficients between bulk indices and bioactivity readouts (n = 15).

| **Variable 1** | **Variable 2** | **n** | **Pearson r** | **p (two-tailed)** |
| --- | --- | --- | --- | --- |
| TPC | ABTS | 15 | 0.0755 | 0.7892 |
| TFC | ABTS | 15 | −0.3641 | 0.1822 |
| TPC | AGI | 15 | 0.4313 | 0.1085 |
| TFC | AGI | 15 | 0.3713 | 0.1731 |

**Notes:** Abbreviations: TPC, total phenolic content; TFC, total flavonoid content; ABTS, ABTS radical scavenging capacity; AGI, *α*-glucosidase inhibition (200 μg/mL).

**Table S2.** Molecular docking scores (ΔG, kcal/mol) of representative phenolic compounds against five core targets.

| kcal/mol | AKT1 (PDB: 8R5K) | RELA (PDB: 6QHL) | AGER (PDB: 3O3U) | TNF (PDB: 2WNV ) | MAPK14 (PDB: 2GFS ) |
| --- | --- | --- | --- | --- | --- |
| Norwogonin | **-7.3** | **-7.5** | **-8.9** | **-10.1** | -7.2 |
| Jaceidin | -6.4 | -6.3 | -8.5 | -9.1 | **-7.3** |
| Gossypetin 3,8,3'-trimethyl ether | -6.2 | -6.9 | -8.6 | -8.4 | -6.9 |
| Genkwanin | -6.7 | -7.3 | -8.7 | -9.9 | -7.1 |
| Azaleatin | -6.6 | -6.8 | -8.7 | -9.4 | -7.2 |

**Notes:** Binding energies were calculated using AutoDock Vina 1.1.2; more negative values indicate stronger predicted binding affinity. All values represent *in silico* predictions and require experimental validation.

**Table S3.** Comparative analysis of the present study with representative published literature on *Psidium guajava* chemical profiling, analytical platforms, and target bioactivity mechanisms.

| **Reference** | **Geographic/ Germplasm Scope** | **Analytical Platform & Depth** | **Metabolite Coverage** | **Target Bioactivities & Mechanisms** | **Key Bioactive Drivers** | **Core Conceptual Advantage** |
| --- | --- | --- | --- | --- | --- | --- |
| (Valdivia-Padilla et al., 2025) | 2 Mexican cultivars (Caxcana & S-56; Calvillo-Cañones region) | GC–MS (Methanol leaf extracts) | 28 identified compounds (3 primary, 25 secondary) | *In silico* target prediction & docking of a single compound (*β*-caryophyllene) against 5 proteins (CB2, PPAR*α*, BAX, BCL2, AKT1) | *β-*Caryophyllene (16.46%–23.06%; sole compound evaluated in modeling) | Focused on 28 GC-MS volatile constituents and single-compound docking across 2 local cultivars, without multi-compound pathway enrichment analysis. |
| (Díaz-de-Cerio et al., 2023) | Single origin (Spanish cultivated leaves from Motril, Spain) | HPLC-ESI-QTOF-MS (80% ethanol leaf extracts) | 73 phenolic compounds (flavonols, flavan-3-ols, gallic/ellagic derivatives, flavanones, 1 anthocyanin) | *In silico* virtual screening via the diabetes-dedicated DIA-DB server (docking & 3D shape similarity) across 18 diabetes mellitus targets | Naringenin (highest multi-target affinity), Quercetin, Catechin, Ellagic acid, Guavinoside C, Guaijaverin, Isoquercitrin | Profiled 73 phenolic constituents from a single geographic origin using DIA-DB computational docking and molecular shape similarity modeling. |
| (Díaz-de-Cerio et al., 2016) | 2 varieties (pomifera & pyrifera), Spain | HPLC-DAD-ESI-QTOF-MS (80% ethanol leaf extracts) | 72 phenolic compounds identified (flavonols, flavan-3-ols, gallic/ellagic derivatives, flavanones; 12 newly reported) | *In vitro* total antioxidant capacity assays via FRAP and TEAC | Flavonols and flavan-3-ols (Guajaverin, Avicularin, Catechin); TPC: var. pyrifera (113.34 mg/g DW) > var. pomifera (86.12 mg/g DW) | Focused on hydroethanolic extraction optimization, LC-MS phenolic profiling of 72 constituents, and inter-variety antioxidant evaluation |
| (Rojas-Garbanzo et al., 2017) | Peel and flesh of pink guava (*Psidium guajava* L*.* cv. 'Criolla') | UHPLC-DAD-MS/MS (90% methanol with 1% formic acid extracts) | 60 phenolics (ellagitannins, flavones, flavonols, flavanols, proanthocyanidins, dihydrochalcones, anthocyanidins; 24 newly reported) | Phytochemical profiling and qualitative characterization of fruit polar compounds | Ellagitannins, flavonols, flavanols (Quercetin-hexosides, Catechin gallate, Phlorizin, Nothofagin); peel vs. flesh distribution | UHPLC-MS/MS profiling of phenolic and polar compounds in pink guava fruit tissues (peel vs. flesh) |
| (Uuh Narvaez et al., 2025) | Edible parts of white guava (*Psidium guajava* L.) fruits harvested in Tixmehuac, Yucatán, Mexico. | UPLC-ESI-MS (Non-targeted UPLC-QTOF-HRMS and targeted dynamic multiple reaction monitoring UPLC-QqQ-MS) | 48 molecules tentatively identified via non-targeted metabolomics; 19 phenolic metabolites and phenylalanine quantified via targeted metabolomics. | *In vitro* *α*-amylase (*α*-AMY, competitive mechanism, IC_50_ = 23.83 mg/mL) and *α*-glucosidase (*α*-GLU, mixed mechanism, IC_50_ = 48.81 mg/mL) inhibitory activities. | (-)-Epigallocatechin (21.07 μg/g DW), (+)-catechin (9.70 μg/g DW), ellagic acid (7.90 μg/g DW), gallic acid (6.58 μg/g DW), and quercetin 3-D-galactoside (5.54 μg/g DW). | Comprehensive targeted/non-targeted metabolic profiling of white guava fruit aqueous extracts and evaluation of their carbohydrate-degrading enzyme inhibition mechanisms. |
| (Zhang et al., 2024) | Peel and flesh of guava fruit cultivar "New Age" from Guangdong Ocean University, Zhanjiang, China. | HS-SPME combined with GC-MS, HPLC, and RNA sequencing (transcriptome analysis). | 90 VOCs, 3 sugars, and 8 organic acids identified. | Flavor formation dynamics, tissue-specific VOC variations across developmental stages, and key metabolic gene expression patterns. | Terpenoids, aldehydes, esters, sugars (fructose, glucose, sucrose), and organic acids; early stages are critical for flesh VOC variation, while peel changes occur progressively. | Characterizing aroma and flavor profiles and underlying molecular mechanisms in fruit peel and flesh during guava development using multi-omics. |
| Present Study | Five native wild accessions across contrasting macro-climates (Amazon Rainforest vs. Semi-arid Lowlands, Bolivia) | Widely targeted LC-MS/MS metabolomics platform | 1,230 phenolic features annotated, with 753 differentially accumulated metabolites (DAMs) prioritized across 10 pairwise comparisons. | Integrated *in vitro* assays (ABTS radical scavenging and *α*-glucosidase inhibition) combined with *in silico* network pharmacology mapping against the complete AGE-RAGE and PI3K/Akt pathways. | Norwogonin, Jaceidin, and tiered-prioritized metabolic markers driving spatial/climatic variations | Established a dual-chemotype framework driven by environmental stress. Discovered an 'aglycone vs. glycoside trade-off'. First identification of Norwogonin and Jaceidin as potent AGE-RAGE modulators. |

References

Díaz-de-Cerio, E., Girón, F., Pérez-Garrido, A., Pereira, A. S. P., Gabaldón-Hernández, J. A., Verardo, V., Segura Carretero, A., & Pérez-Sánchez, H. (2023). Fishing the Targets of Bioactive Compounds from *Psidium guajava* L. Leaves in the Context of Diabetes. *International Journal of Molecular Sciences*, *24*(6). <https://doi.org/10.3390/ijms24065761>

Díaz-de-Cerio, E., Gómez-Caravaca, A. M., Verardo, V., Fernández-Gutiérrez, A., & Segura-Carretero, A. (2016). Determination of guava *(Psidium guajava* L.) leaf phenolic compounds using HPLC-DAD-QTOF-MS. *Journal of Functional Foods*, *22*, 376-388. <https://doi.org/https://doi.org/10.1016/j.jff.2016.01.040>

Rojas-Garbanzo, C., Zimmermann, B. F., Schulze-Kaysers, N., & Schieber, A. (2017). Characterization of phenolic and other polar compounds in peel and flesh of pink guava (*Psidium guajava* L. cv. ‘Criolla’) by ultra-high performance liquid chromatography with diode array and mass spectrometric detection. *Food Research International*, *100*, 445-453. <https://doi.org/10.1016/j.foodres.2016.12.004>

Uuh Narvaez, J. J., Guerrero-Analco, J. A., Monribot-Villanueva, J. L., Chan-Zapata, I., & Segura Campos, M. R. (2025). Metabolic profile of *Psidium guajava* L. fruits and their inhibitory properties on *α*-amylase and *α*-glucosidase. *Fitoterapia*, *185*, 106781. <https://doi.org/10.1016/j.fitote.2025.106781>

Valdivia-Padilla, A., Sharma, A., Zegbe, J., & Morales-Domínguez, J. (2025). Metabolomic Characterization and Bioinformatic Studies of Bioactive Compounds in Two Varieties of *Psidium guajava* L. Leaf by GC–MS Analysis. *International Journal of Molecular Sciences*, *26*(6). <https://doi.org/10.3390/ijms26062530>

Zhang, J., Zhang, Y., Zou, S., Yang, E., Lei, Z., Xu, T., & Feng, C. (2024). Characterization of the aroma and flavor profiles of guava fruit (*Psidium guajava*) during developing by HS-SPME-GC/MS and RNA sequencing. *Food Chemistry. Molecular Sciences*, *9*, 100228. <https://doi.org/10.1016/j.fochms.2024.100228>
